# Effect of Gaseous Citral on Table Grapes Contaminated by *Rhizopus oryzae* ITEM 18876

**DOI:** 10.3390/foods11162478

**Published:** 2022-08-17

**Authors:** Laura Quintieri, Francesco Fancello, Leonardo Caputo, Andrea Sorrentino, Severino Zara, Vincenzo Lippolis, Salvatore Cervellieri, Francesca Fanelli, Antonia Corvino, Bernardo Pace, Maria Cefola

**Affiliations:** 1 Institute of Sciences of Food Production, National Research Council of Italy (CNR), 70126 Bari, Italy; 2 Department of Agricultural Sciences, University of Sassari, 07100 Sassari, Italy; 3 Institute of Polymers, Composites and Biomaterials (IPCB), National Research Council of Italy (CNR), 80055 Portici, Italy; 4 Institute of Sciences of Food Production, National Research Council of Italy (CNR), c/o CS-DAT, 71121 Foggia, Italy

**Keywords:** citral, post-harvest, table grape decay, spoilage, monoterpene, natural antimicrobials, preservation strategy

## Abstract

*Rhizopus oryzae* is responsible for rapidly producing a deliquescent appearance in grape berries, generally favoured by cold chain interruptions. To counteract fruit spoilage and to meet consumer acceptance, innovative strategies based on the application of natural compounds are ongoing. Due to their biological activities, including antimicrobial ones, natural flavour compounds extend the shelf life and improve the nutritional value as well as the organoleptic properties of foods. Thus, in this work, the application of the antimicrobial citral, a flavor component of monoterpenes identified in plant and fruit essential oils, was developed and validated against one spoiler of *R. oryzae*. Citral, as pure compound, was first investigated in vitro against *R. oryzae* ITEM 18876; then, concentrations equal to the minimal inhibitory concentration (MIC) and 4-fold MIC (4MIC) value were applied on the table grape cv Italia infected with this strain and stored. The MIC value was equal to 0.0125 μL/cm^3^; both citral concentrations (0.0125 and 0.05 µL/cm^3^) were effective in counteracting the microbial decay of infected table grapes over the storage period. The HS-SPME/GC-MS method showed citral persistence in the head space of plastic trays with the infected samples; as expected, a higher content of citral isomers was found in the sample treated with 4MIC value. In conclusion, citral revealed its efficacy to counteract the onset of soft rot by *R. oryzae* ITEM 18876 under storage conditions. Thus, it could be successfully exploited to develop an active packaging or natural preservatives to extend table grape shelf life without affecting its quality and sensory characteristics, whilst also satisfying the consumer demand for natural preservative agents.

## 1. Introduction

Table grape (*Vitis vinifera* L.) is a non-climateric fruit with serious problems during post-harvest handling, storage and marketing due to the growth of undesired microorganisms [1]; among these, *R. stolonifer* and *R*. *oryzae* (also named *R. arrhizus*) cause post-harvest soft rot [2,3]. *Rhizopus* rot usually starts at the base of the pedicel in mature berries, causing first a water-soaked appearance in fruit tissues; then, a brownish-gray to blackish-gray mold develops along the fissures and even on rachis, leading to the breakdown of infected berries and juice lost. Rots are often associated with injured berries under warm weather conditions during harvest [3,4]. *Rhizopus* spp. can also cause post-harvest losses of table grapes without apparent symptoms; however, when a light pressure is applied to infected berries (e.g., as in the case of densely packed bunches), the rot can come out [5].

Product decay by *Rhizopus* spp. under cold storage conditions cannot be excluded; indeed, although the temperature needs to be optimal and constant, especially for long distance shipment, accidental temperature fluctuations along the cold chain can occur, favoring the development of microbial decay. Unsurprisingly, strategies to improve the traceability and transparency of table grapes’ cold chain logistics are ongoing [6,7].

In addition to preservation at low temperature, table grape producers and retailers are constantly searching for innovative technologies able to both reduce the use of fungicides and counteract the physicochemical changes occurring during long-term storage in order to maintain optimal quality parameters during product marketability [8].

Considering the high susceptibility to plant diseases and parasites, grapes and especially table grapes are subject to numerous treatments of different pesticides (up to 18 species per biological cycle). In particular, table grapes are usually treated during pre- and post-harvest processes with cyprodinil + fludioxonil (Switch, Syngenta Italia, Milan, Italy), difenoconazole (Score^®^ EC 25, Syngenta Italia, Italy), Boscalid 50 WG (Cantus BASF, Ludwigshafen, Germany), Pyrimethanil (Scala^®^, BASF, Ludwigshafen, Germany), Fludioxonil (Geox^®^, Syngenta, Basel, Switzerland), Fenexamide (Teldor plus^®^, Bayer, Leverkusen Germany), Fluazinam (Banjo^®^, Makhteshim Agan Italia, Grassobbio, Italy ), and Iprodione (Rovral^®^, BASF Ludwigshafen, Germany) [9,10]. Sulfur dioxide (SO_2_) generators are also used to control rots on post-harvest table grapes, but the unsuitable application of this gas may induce injuries and unpleasant aftertaste in both rachis and berries [11]. In addition, sulfite residues are becoming an important consumer issue for adverse reactions; the current amount of SO_2_ permitted by the Regulation 1333/2008 on table grapes is 10 mg/Kg [12].

In accordance with consumer demands, natural molecules endowed with antimicrobial properties and green strategies are widely studied to maintain the acceptability of fresh foods during storage as alternatives to synthetic preservatives [13,14,15,16,17,18]. Due to both the ability to counteract microbial spoilage and improve organoleptic properties, flavor compounds identified in essential oils (EOs) have been widely exploited in food applications [13,19]. Plant essential oils constituents are mainly grouped into two categories, oxygenated compounds (alcohols, esters, ketones, phenols, and other aromatic and sulfur-containing groups), and hydrocarbons. These latter are characterized by a specific chemical group terpenoids, in turn composed by monoterpene hydrocarbons, sesquiterpene hydrocarbons, and their oxygenated derivatives [20].

Among identified terpenes in EOs, the acyclic monoterpenoid citral (consisting of two geometrical isomers: the E-isomer geranial and the Z-isomer neral [21]) is the most widely used flavouring compound in foods, beverages, and cosmetics with a high consumer acceptance [22]. It is classified by the European Commission among the compounds approved as flavor additives in food, beverages [23], and feed products [24]. Due to its antimicrobial properties, citral is also studied for applications in novel and natural preservative strategies [21,25,26,27]. However, relatively few investigations of citral-based strategies have been performed to preserve table grapes’ quality and safety during post-harvest storage; essential oils of *Eucaliptus staigeriana*, composed by 30.9% citral, were able to reduce the incidence and severity of grey rot caused by *Botrytis cinerea* and the severity of ripe rot caused by *Colletotrichum acutatum* on table grapes [28]. Similarly, lemongrass oil, containing citral and geraniol, was used to develop nanoemulsion coatings to preserve grape berries’ quality and safety [29]. To the best of our knowledge, no studies have been, instead, performed to investigate citral’s effect, as a pure compound, on the control of table grape soft rot under storage conditions.

In light of these considerations, in this work, the antimicrobial activity of citral, as a pure compound, is, firstly, investigated in vitro against the spoiler *R. orizae* ITEM 18876 to find the minimal inhibitory concentration (MIC); then, for the first time, the antimicrobial effect of citral is demonstrated on table grape cv. Italia bunches infected with the same strain and kept under sub-optimal storage conditions in order to both favor rot and simulate cold chain breakage.

## 2. Materials and Methods

### 2.1. Chemicals and Reagent

Chemicals, standards, and reagents for post-harvest quality analysis were purchased from Sigma-Aldrich (St. Louis, MO, USA), whereas microbiological media were obtained from Biolife Italiana srl (Milan, Italy). Methanol (HPLC grade) and citral (C_10_H_16_O, CAS number: 5392-40-5) (≥96%, Food Chemicals Codex, FCC, natural, food grade, FG) were purchased from Sigma-Aldrich (Milan, Italy).

### 2.2. Culture Conditions

The *R. oryzae* used in this study was ITEM 18876 from the Agro-Food Microbial Culture Collection of the Institute of Sciences of Food Production (ISPA-CNR) (http://www.ispa.cnr.it/Collection accessed on 17 January 2022). This strain was routinely cultured on potato dextrose agar (PDA) and incubated at 25 °C for 7 days to induce sporulation. At the end of incubation period, the cultures were covered with 5 mL of a sterile saline solution supplemented with Tween 80 (0.1 mL/L) and gently rubbed with a sterile cotton swab; then, the conidia suspension was transferred to a sterile tube and filtered (11 µm, Nylon Net Filtres, Millipore, Cork, Ireland). Spore concentration was determined by the spore-counting method in a Thoma chamber. Serial dilution in a sterile saline solution (0.9% NaCl) of this suspension were performed to obtain the inocula for each experiment.

### 2.3. In Vitro Antimicrobial Activity and Minimal Inhibitory Concentration (MIC) Determination

The in vitro antimicrobial activity of citral against *R. oryzae* ITEM 18876 was evaluated by using both the disk diffusion method and the disk volatilization method [30]. Two inocula of 10^6^ spores/mL and 10^7^ spores/mL, obtained as reported above, were used for each assay.

In brief, the disk diffusion method was performed by spreading 100 µL of each inoculum on PDA Petri dishes; then, a sterile paper disk (Whatman No. 1, diameter 6 mm) soaked with a citral amount in the range from 0.5 µL to 4 was laid on the surface of the inoculated plate and incubated for 7 days at 25 °C. Disks soaked with sterile water were included as controls. At the end of the incubation, the inhibition activity of citral was observed as clear halos around the disks.

The disk volatilization method followed the procedure described by Fancello et al. [25] and a citral concentration ranging from 0.006 to 0.1 μL/cm^3^. In brief, PDA agar was inoculated with each spore suspension inoculum. Sterile paper disks were glued to the cover of each Petri plate and then soaked with each of the citral concentrations tested; then, the plates were sealed with parafilm and incubated at 25 °C for 7 days.

The MIC was expressed as microliters of citral per volume of head space above the inoculated agar surface (µL/cm^3^) and defined as the lowest vapor concentration that did not produce a clearly visible growth. Each test was performed in triplicate and the experiments were repeated twice.

### 2.4. Antimicrobial Activity of Gaseous Citral on Inoculated Table Grape Berries

Healthy table grape cv Italia, purchased from a local retail grocer (Sassari, Italy), were transported to the laboratory and immediately processed as described in this paper. Grape bunches were firstly washed with sterile distilled water, disinfected for 3 min in a hypochlorite solution (2.5 %, *v*/*v*), and then washed twice with distilled sterile water. After drying for 30 min in a sterile tray, single berries were detached; 5 µL of 10^6^ spores/mL were inoculated in the wound formed by pedicel removal and the berries were placed in a sterile reservoir (VWR, Milan, Italy). To assess the effect of gaseous citral on the growth of *R*. *oryzae* ITEM 18876, six plastic trays containing each 6 inoculated berries were prepared; then, 3 reservoirs were placed inside 20 L sealed polyethylene box, equipped with a circulation fan and a heating system, which allow the citral to spread and saturate the box volume [25]. The other 3 reservoirs (positive control) were not exposed to the citral vapor and they were placed inside of plastic trays covered with a lid (34 × 23 × 16 cm, Gensini, Florence, Italy). Citral treatments were performed using two concentrations, the first equal to the MIC and the second one equal to 4-fold the MIC value (4MIC); to favor the volatilization of citral, the circulation fan and heating system were left on for 2 h.

Additional six trays containing 6 un-inoculated berries each as negative control were also prepared each as negative control. Three were placed inside the polyethylene box and the other three in a plastic tray with lid (34 × 23 × 16 cm, Gensini, Florence, Italy). The box and plastic tray were incubated at 25 °C for 7 days and the mycelia growth was observed daily.

### 2.5. Post-harvest Evaluation of Table Grapes under Citral Treatment

#### 2.5.1. Sample Packaging and Storage

One set of 27 clusters of organic table grapes (ca. 200 g) with apparently healthy berries were selected and disinfected with a 100 ppm chlorine solution (adjusted to pH 7.0) for 1 min. Then, all clusters were washed twice with sterile distilled water and allowed to dry spread on filter papers for 30 min.

Each bunch was externally sprayed with ca. 5–6 mL of inoculum containing 10^3^–10^4^ spores/mL of *R. oryzae* ITEM 18876. Subsequently, the bunches were dried as above and, then, transferred to a plastic tray (5 × 10 × 5 cm). An opened 25 mL plastic jar (VDSOW, China) containing a 200 mg cotton ball (Cottonplus, Italy), impregnated with the two citral concentrations (MIC and 4MIC), was placed on one side of the sample tray. Citral-untreated samples sprayed with saline solution were included as controls. Then, treated and untreated sample trays were individually transferred into a plastic bag (18 × 28 cm) (HDPE Cooki Gelo più, Cooki Cofresco SpA, Volpiano, Italy). The samples were closed without sealing at the edges and stored at 12 °C and 80–85% relative humidity for 5, 10, and 15 days as previously reported [31]. Three replicates of trays were prepared for each storage time.

#### 2.5.2. Decay Incidence Determination

For each treatment and sampling day, decays occurred on infected and stored grape clusters were visually assessed. The decay incidence was calculated using the formula [32]:

(1) $$\mathrm{Decay}\;\mathrm{incidence}\;\left(\%\right)=\left(\frac{\mathrm{Number}\;\mathrm{of}\;\mathrm{decayed}\;\mathrm{bunches}\;}{\mathrm{Total}\;\mathrm{number}\;\mathrm{of}\;\mathrm{bunches}}\right)\times 100$$

Grape clusters were considered “decayed” when at least the 5% of berries were moldy (≥3 in the European and Mediterranean Plant Protection Organization scale [33]).

#### 2.5.3. Visual Quality and Perception of Citral Odor

Visual quality (VQ) and the sensory perception of citral odor in table grape samples were evaluated by 10 panelists at each sampling day (0, 12, and 15), according to the scoring rating scale reported by [34]. VQ was evaluated using a hedonic scale from 5 to 1 (5 = excellent; 4 = good; 3 = fair, limit of sensory acceptability; 2 = poor; 1 = very poor), whereas the sensory perception of citral odor in each sample was scored on a rating scale from 1 to 5 (1 = absence, 2 = light; 3 = moderate; 4 = severe; 5 = extreme).

#### 2.5.4. Determination of Citral by GC-MS Analysis

The determination of (E)- and (Z)-citral was carried out by a headspace solid-phase microextraction (HS-SPME) coupled to gas chromatography–mass spectrometry (GC-MS). Specifically, a GC-MS system composed by an Agilent 6890A GC (Agilent Technologies, Palo Alto, CA, USA) coupled to an Agilent 5973N inert MSD mass spectrometer equipped with Triple-Axis HED-EM detector was used. Two plastic trays were used for the citral analysis containing table grape samples and a plastic capsule impregnated with two citral concentrations (MIC and 4MIC). Determinations were carried out at different sampling days (0, 1, 7, and 15). For citral extraction and desorption, a HS-SPME procedure was performed using a manual SPME sampler holder (Supelco, Bellafonte, PA, USA). For analyte extraction, a SPME divinylbenzene/carboxen/polydimethylsiloxane (DVB/CAR/PDMS, 1 cm fiber length) fiber was exposed to the plastic tray headspace for 30 min at 12 °C. After the extraction, headspace volatiles were thermally desorbed exposing the fiber into the Split/Splitless Injection Port of GC-MS system, which was equipped with a 0.75 mm i.d. Ultra-Inert Liner Straight and was kept at 250 °C for 5 min in splitless mode. The citral analysis was carried out by the GC-MS system equipped with a VF-WAXms (60 m × 0.25 mm i.d., 0.25 μm film thickness, Agilent Technologies) fused-silica capillary column and using programmed temperature mode. In particular, the temperature program started at 40 °C and held for 5 min, then raised at the rate of 0.033 °C/s to 140 °C, then raised at the rate of 0.083 °C /s to 170 °C, then raised at the rate of 0.833 °C/s to 230 °C, and held for 10 min. The helium flow was set to 0.016 × 10^−3^ L/s. The transfer line, ion source, and quadrupole temperatures were 280, 290, and 150 °C, respectively. Electron impact Ionization (EI+) mode with an electron energy of 70 eV was used and the mass spectra were recorded in the m/z range of 40–300 u. The total chromatographic run time was 72.2 min. The identification of the two isomeric forms of citral, (Z)-citral (retention time, tr = 53 min) and (E)-citral (tr = 56 min), was carried out by comparing the sample mass spectra with the spectra in the NIST/EPA/NIH Mass Spectral Database (National Institute of Standards and Technology, Version 2.0 f, 2008, Gaithersburg, MD, USA), using a match quality higher than 80. To confirm their identity, the linear retention index (LRI) was calculated for each volatile compound in relation to the retention times of C5–C29 n-alkanes and compared with those reported in the literature [35,36]. The evaluation of the concentration of the (E)- and (Z)-citral was carried out normalizing the measured peak areas to their peak areas at the first sampling (t = 0), which was assumed to be equivalent to 100%.

#### 2.5.5. Microbiological Analysis

At 0, 5, 10, and 15 days of storage 100 g of apparently intact berries were selected and homogenized for 5 min in a Stomacher Lab Blender 400 (Seward Ltd., Worthing, West Sussex, UK). A total of 25 grams of the homogenized grape sample were transferred into 90 mL of the sterile physiological saline solution (9 g/L NaCl) and homogenized for 2 min as above. Then, 100 μL of each homogenate was plated in duplicate on PDA for counting *R. oryzae* ITEM 18876 after 16–18 h of incubation at 25 °C under aerobic conditions.

### 2.6. Statistical Analysis

The results of post-harvest trials and microbiological analysis were processed using the IBM-SPSS software package v.25.0 for Windows. A one-way analysis of variance (ANOVA) was also performed to determine the significance of mean differences in decay incidence and microbiological counts among samples, using HSD Duncan’s test to examine if the differences were significant at *p* < 0.05.

## 3. Results and Discussion

### 3.1. In Vitro Antimicrobial Assay

A preliminary evaluation of citral antimicrobial activity was performed against *R. oryzae* ITEM 18876 by applying the disk diffusion method at two inoculum levels and different citral concentrations (from 0.5 µL to 4 of pure citral). At the optimum growth temperature (25 °C) of incubation, the results show that the lowest concentration used (0.5 µL) completely inhibited *R. oryzae* ITEM 18876 growth at both levels of inocula (see Supplementary Materials Figure S1). These data are in accordance with other studies reporting the inhibitory activity of citral against fungi, including *R. stolonifer* [37,38]; however, a higher active concentration was reported against the latter [37]. Overall, the monoterpene citral exhibited a broad antimicrobial spectrum against bacteria and fungi; MIC values ranging from 50 to 1000 µg/mL were registered by other authors [27]. 

Studies on *Penicillium digitatum* showed that one of the fungicidal mechanisms exerted by citral is represented by the repression of five ergosterol biosynthetic genes, leading to cell membrane damage and mycelial growth inhibition [39]. Additionally, for *Rhizopus* spp., the fungicidal mechanism of citral was proposed to act by reducing the ergosterol content in the plasma membrane [22].

The antimicrobial activity of citral against different bacteria and plant pathogenic fungi in functionalized coatings [27,40,41,42,43,44], spray dried microparticles [45], and nanostructured lipid carriers was also demonstrated [46]. In particular, the incorporation of the volatile antimicrobial citral into packaging materials has the advantage that it can exert an antimicrobial activity without physical contact with the food surface; the elevated vapor pressures allow citral to be released into the headspace, subsequently, and be absorbed by the food matrix [47,48].

Thus, considering the previous results, the antifungal activity of citral against the target strain was evaluated in relation to the citral levels in the headspace under the experimental conditions. The results show that the MIC value for citral vapor against *R. oryzae* ITEM 18876 was equal to 0.0125 μL/cm^3^ for the two different assayed spore concentrations. In a previous study, a concentration equal to 0.06 μL/mL inhibited only one-third of the radial mycelial growth of *Penicillium italicum*, whereas higher concentrations (>0.5 μL/mL) caused the full inhibition or fungicidal activity of the same fungus displaying a MIC of 0.5 μL/mL [49].

### 3.2. Antimicrobial Activity of Gaseous Citral in Food Matrix

Aiming at scouting and validating the optimal citral concentration to be assayed in post-harvest trials, 0.0125 and 0.05 µL/cm^3^ of citral amounts (corresponding to MIC and 4 MIC) were preliminary tested on table grape berries infected with *R. oryzae* ITEM 18876 under conditions favoring fungal growth; the 4MIC value was selected considering the food matrix that could affect the antimicrobial effects. Citral is a low toxic molecule and these concentrations are considered safe for human health [50].

**Figure 1:**
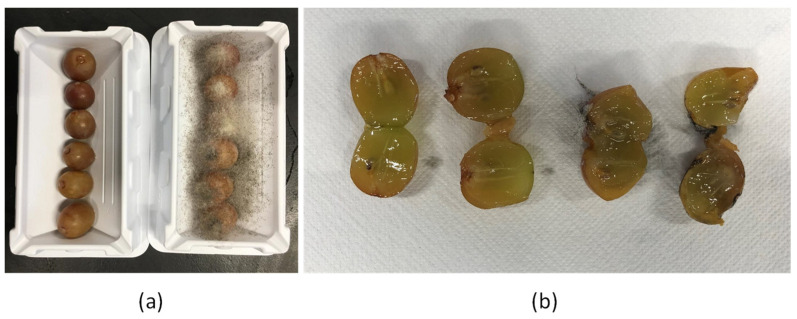
Table grapes artificially contaminated with *R. oryzae* ITEM 18876 (10^6^ spores/mL) and incubated under citral exposure (0.0125 µL/cm^3^) for seven days at 25 °C. (**a**) Comparison between the inoculated treated (**left**) and not-treated (**right**) grape berries. (**b**) A section of the grape berries showing structure changes between the inoculated treated (**left**) and not-treated (**right**) samples.

These results confirmed the efficacy of the selected citral concentration in controlling ITEM 18876 soft rot appearance on inoculated berries. Indeed, after seven days of incubation at 25 °C, the treated berries did not show mycelium occurrence and spoilage symptoms (Figure 1a), except for a slight the browning registered in the untreated samples and presumably due to the increased temperature during the assay. As shown in Figure 1b, differences in the berries’ structure were observed in both samples; a slight softening in the treated berries was also observed.

### 3.3. Post-Harvest Evaluation of Citral-Treated Table Grapes during Storage

The sensory evaluation of VQ was not significantly affected by citral treatment on each storage day, although, during the experiment, a significant reduction in VQ was scored in all treatments, as a consequence of decay. As for the citral odor perception in fruits, it was highly perceived in the treated fruits after 15 days (mean score of about 4), whereas, at the end of storage, a lower odor score was attributed (mean score around 3) to the treated samples. Anyway, the perception of citral was notable only after the opening of the bags, after which (around 30 min at air) the citral odor was lost.

Regarding the determination of citral in the head space of the plastic trays containing the table grape samples, the HS-SPME/GC-MS method allowed us to evaluate the concentrations of two isomeric forms of citral, i.e., (Z)-citral and (E)-citral, in the head space of the plastic trays containing the grape samples. A reduction in the content of (Z)-citral and (E)-citral of 62 and 58%, respectively, was observed after only 1 day of storage for the samples treated with 0.0125 µL/cm^3^. An almost total reduction (99%) in both molecules was registered after 15 storage days, even if their presence was perceived by the panelists because of the gas diffusion into the plant tissue. In the case of samples treated with 0.05 µL/cm^3^, a lower reduction in citral content with respect to samples treated with 0.0125 µL/cm^3^ was observed. Specifically, a reduction in (Z)-citral of 27, 40, and 79% at 1, 7, and 15 sampling days was measured. Similarly, it was observed a decrease in (E)-citral of 29, 56, and 83% on the same sampling days. These results indicate that, by using 0.05 µL/cm^3^, a higher content of both isomers of citral was found in the head space of the used plastic tray. It is worth underlining that citral was found as one of the main compounds detected in seedless grapes at the end of ripening [51] and this could increase the acceptability by the consumer. However, the sensory attributes of other fruits stored in packaging functionalized with citral were not affected by treatment [52,53].

**Figure 2:**
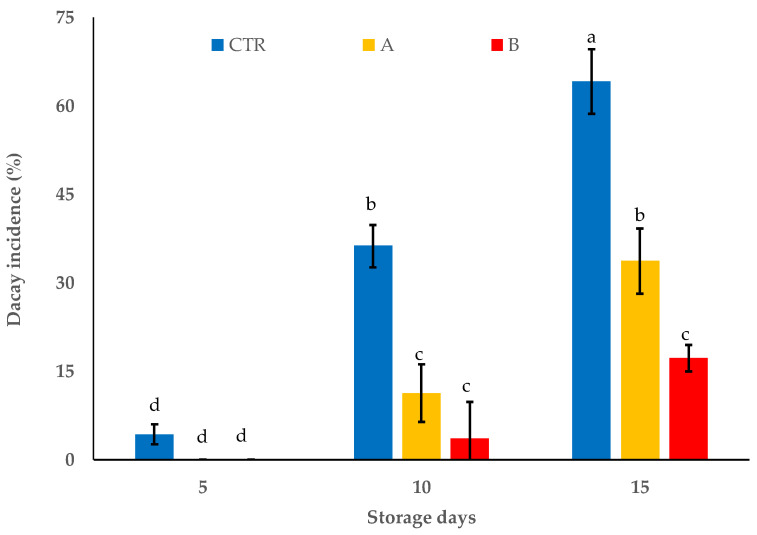
*R*. *oryzae* ITEM 18876 decay incidence percentages on the inoculated table grapes (cv. Italia) packed in HDPE bags with different citral doses (A equal to MIC value of 0.0125 µL/cm^3^ and B equal to 4-fold MIC value of 0.05 µL/cm^3^, respectively) and stored at 12 °C and 85% relative humidity for 15 days. CTR: control without citral. Bars are means ± standard deviations (*n* = 3). Bars with different lowercase letters represent significantly different averages (*p* < 0.0001; HSD Tukey’s test; *F*(8.18) = 89.297, *p* = 6.732 × 10^−13^).

**Figure 3:**
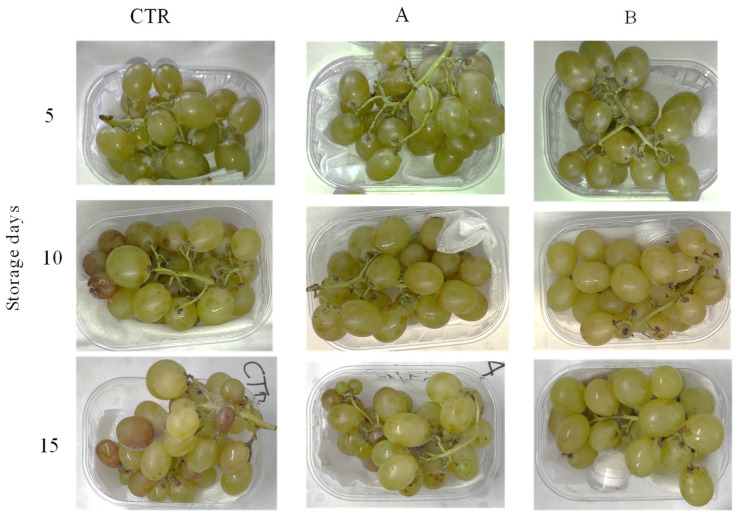
Inoculated table grapes (cv. Italia) packed in HDPE bags with different citral doses (A equal to MIC value of 0.0125 µL/cm^3^ and B equal to 4-fold MIC value of 0.05 µL/cm^3^, respectively) and stored at 12 °C and 85% relative humidity for 15 days. CTR: control without citral. Bars are means ± standard deviations (*n* = 3).

Overall, the decay incidence recorded in the treated samples was significantly lower than that of the control grape samples (*p* < 0.05), regardless of the applied citral doses (Figure 2 and Figure 3). Starting from day 10 of storage, in berries treated with 0.0125 µL/cm^3^, rotting increased by reaching 25.5% of incidence, on average, at the end of the trial; the treatment with the highest citral dose resulted in values significantly (*p* < 0.05) lower than those registered with the lowest dose (incidence of rot by 3.6 and 17.2% at days 10 and 15 of incubation, respectively) (Figure 2). By contrast, the control sample showed rotting that linearly increased with storage time and reached average decay percentages of 64.2% already from day 5. Quite similar values were previously reported in table grapes treated with modified atmosphere packaging (MAP) in combination with eugenol or thymol concentrations higher than those used in the present work [54].

**Figure 4:**
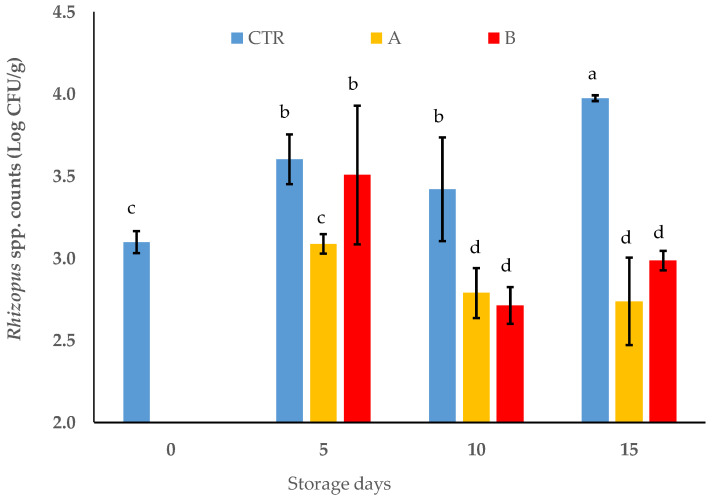
*R*. *oryzae* ITEM 18876 counts on the inoculated table grapes (cv. Italia) packed in HDPE bags with different citral doses (A equal to MIC value of 0.0125 µL/cm^3^ and B equal to 4-fold MIC value of 0.05 µL/cm^3^, respectively) and stored at 12 °C and 85% relative humidity for 15 days. CTR: control without citral. Bars are means ± standard deviations (*n* = 9). Bars with different lowercase letters represent significantly different averages *(p* < 0.0001; HSD Tukey’s test; *F*(9.80) = 38.050, *p* = 2.549 × 10^−25^).

As expected, *R. oryzae* ITEM 18876 growth in the untreated samples showed slight increments at 12 °C (Figure 4); however, the counts of *R. oryzae* ITEM 18876 on the inoculated grape clusters and alternatively treated with the two citral doses were, on average, lower than those registered for the control clusters over the period of incubation. Indeed, simple main effects showed that both doses of citral had a higher and significant (*p* < 0.001) inhibitory effect on *R. oryzae* viability starting from day 10 in comparison to the control samples (Figure 4).

In addition to that demonstrated for other fruits [55], our results confirm the efficacy of the low doses of citral also in counteracting *R. oryzae* spoilage in table grapes under temperature conditions higher than the usually used to preserve the product and simulating an accidental cold chain breakage. Citral was also more effective than other molecules identified in EOs to preserve grape quality; only the combination of modified atmosphere packaging (MAP) and amounts of eugenol, thymol, or menthol (0.5 mL) higher than those used in this paper for citral successfully counteracted the microbial decay of grape samples (cv. Crimson Seedless) [56].

Recent efforts in “green technology” development aimed at replacing conventional and not eco-friendly methodologies have allowed the increase in yields and the stability of EOs and their components, such as citral [57,58]; thus, taking into account the results presented in this paper, the latter could be successfully applied in the development of antimicrobial and or edible packaging without economic disadvantages and under a circular economy. The formulation of natural food ingredients that are alternative to synthetic compounds used to preserve grapes’ shelf life is also a feasible and reliable process.

## 4. Conclusions

In conclusion, to the best of our knowledge, the application of low concentrations of gaseous citral was investigated and validated on table grapes during storage in this paper for the first time. The treatment with citral at both assayed concentrations, properly vaporized through suitable and passive diffusers, proved to be very effective in containing the development of soft rot caused by *R. oryzae* ITEM 18876 without affecting the quality and sensory characteristics of the product. Thus, the application of low and non-toxic citral amounts could be used directly in the individual packs of table grapes, maintaining its antifungal efficacy even during domestic refrigeration.

Overall, this natural compound could be successfully exploited in the development of control strategies to extend table grapes’ shelf life, whilst also satisfying the consumer demand for natural preservative agents as well as meeting their acceptance.

## Data Availability

Data is contained within the article or Supplementary Materials.

## Supplementary Materials

The following supporting information can be downloaded at: https://www.mdpi.com/article/10.3390/foods11162478/s1. Figure S1. Disk diffusion method displaying the antimicrobial activity of citral (0.5 µL) against *R. oryzae* ITEM 18876 inoculated at 10^7^ and 10^6^ spores/mL on PDA and incubated for 7 days at 25 °C of incubation.
