# Supplementary material for: Effect of Gaseous Citral on Table Grapes Contaminated by Rhizopus oryzae ITEM 18876
Source: Foods. 2022 Aug 17;11(16):2478. doi: 10.3390/foods11162478 (PMC9407198; doi:10.3390/foods11162478)
Supplement: Supplementary file 1 [file foods-11-02478-s001.zip › foods-1835297-supplementary.pdf]

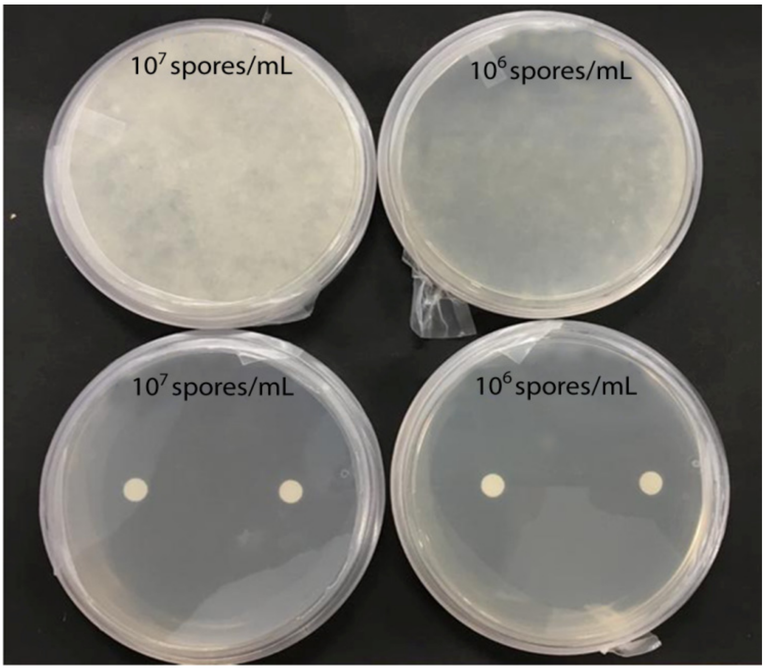

Figure S1. Disk diffusion method displaying antimicrobial activity of citral (0.5 μL) against *R. oryzae* ITEM 18876 inoculated at 10^7 and 10^6 spores/mL respectively on PDA and incubated for 7 days at 25 °C of incubation.
